# *miR-989* Is Required for Border Cell Migration in the *Drosophila* Ovary

**DOI:** 10.1371/journal.pone.0067075

**Published:** 2013-07-03

**Authors:** Jan-Michael Kugler, Ya-Wen Chen, Ruifen Weng, Stephen M. Cohen

**Affiliations:** 1 Institute of Molecular and Cell Biology, Singapore, Singapore; 2 Department of Biological Sciences, National University of Singapore, Singapore, Singapore; University of Toronto, Canada

## Abstract

microRNAs (miRNAs) are small non-coding RNAs that regulate gene expression by destabilizing target transcripts and/or inhibiting their translation. miRNAs are thought to have roles in buffering gene expression to confer robustness. miRNAs have been shown to play important roles during tissue development to control cell proliferation, differentiation and morphogenesis. Many miRNAs are expressed in the germ line of *Drosophila*, and functions have been reported for a few miRNAs in maintenance of stem cell proliferation during oogenesis. Here, we analyse the function of *Drosophila miR-989* in oogenesis. *miR-989* is abundant in ovaries. Mutants lacking *miR-989* did not display gross abnormalities affecting egg chamber formation or maturation. However, the migration of the border cell cluster was severely delayed in *miR-989* mutant egg chambers. We demonstrate that *miR-989* function is required in the somatic cells in the egg chamber, not in germ line cells for border cell migration. Loss of *miR-989* from a fraction of the border cell cluster was sufficient to impair cluster migration as a whole, suggesting a role in border cells. Gene ontology analysis reveals that many predicted *miR-989* target mRNAs are implicated in regulating cell migration, cell projection morphogenesis, cell adhesion as well as receptor tyrosine kinase and ecdysone signalling, consistent with an important regulatory role for *miR-989* in border cell migration.

## Introduction

miRNAs are small non-coding RNAs that function as regulators of gene expression in a wide range of biological contexts [1], [2]. miRNAs associate with their target transcripts via partial complementary base pairing to target sites which are usually located in the target 3'UTR or in coding sequences [3], [4]. In general, miRNAs act as negative regulators of gene expression at the post-transcriptional level by promoting target transcript destabilization and/or by reducing their translation [1], [2].

**Figure 1:**
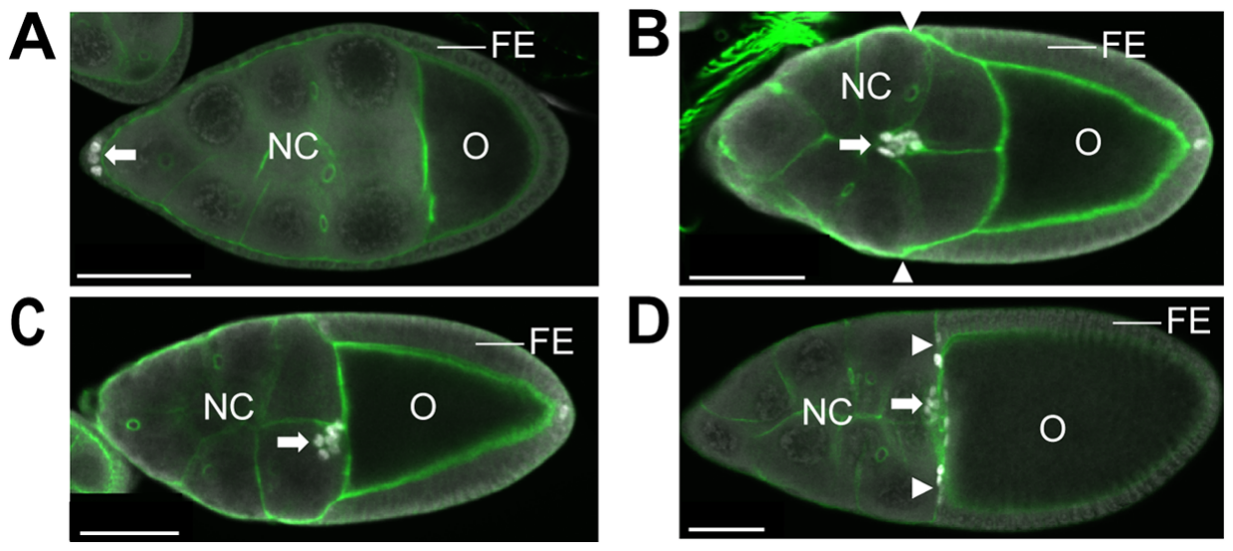
Morphology of mid-oogenesis egg chambers and border cell migration. Mid-oogenesis egg chambers labelled with Phalloidin (green) and border cell marker α-Slbo (white). The germ line derived nurse cell (NC) cluster and oocyte (O) as well as the somatic follicular epithelium (FE), which encapsulates the germ line cells, are identified. A Stage S8 egg chamber. Slbo-positive border cells form in the FE anterior to the NC cluster (arrow). B Stage S9 egg chamber. The FE migrates towards the oocyte where it forms a columnar epithelium. Follicle cells stretch over the NC cluster to form a flat epithelium. The border cells (arrow) migrate through the NC cluster, roughly in parallel to the leading edge of the migrating external follicle cell sheet (arrowheads). C Stage S10A egg chamber. Migration of the border cell cluster and the migrating FE have essentially completed. D Stage S10B egg chamber. The centripetal follicle cells migrate over the anterior face of the oocyte (arrowheads).

Border cells serve as a model system for the study of collective cell migration during *Drosophila* oogenesis [5], [6], [7]. *Drosophila* eggs mature in compound entities called egg chambers, which are comprised of 16 interconnected germ-line cells that are encapsulated by a monolayer of somatic follicle cells [8] (Fig. 1). One of the 16 germ-line cells differentiates as the oocyte, while the other 15 become polyploid nurse cells, which produce RNAs, proteins and organelles for incorporation into the oocyte to aid its maturation. The somatic follicle cells undergo a complex developmental and morphogenetic program that is tightly linked to germ line development and ultimately leads to the formation of the egg shell [7]. A subset of follicle cells, called border cells, has a special role during oogenesis, which involves an invasive, directed, cell migration. During stage 8 of oogenesis the border cells are specified at the anterior pole of the follicular epithelium and start to express the C/EBP transcription factor, Slow border cells (Slbo; Fig 1A). The border cells detach from the follicular epithelium and migrate as a cluster toward the oocyte during stage 9 to 10A (Fig. 1B, C). At stage 10B, the border cell cluster has reached the anterior face of the oocyte and migrates laterally to its anterodorsal position (Fig. 1D). Specification of the border cells and the transition to coordinated cell migration involve several conserved signalling pathways and extensive remodelling of the cytoskeleton and cell adhesion properties [5], [6], [7]. The JAK/STAT pathway is required for border cell specification and for migration [9], [10], [11]. Ecdysone signalling regulates the timing of border cell specification [12], [13], [14]. Within the border cells, the receptor tyrosine kinases EGFR and PVR interpret guidance cues produced by the oocyte to direct anterior migration and later dorsal migration of the cluster [15], [16]. Homophilic adhesive interactions between border cells and the nurse cells involving Cadherins are crucial for normal cluster migration [17].

In this report, we identify the miRNA *miR-989* as a regulator of border cell migration. We show that border cell migration is delayed in *miR-989* mutant egg chambers, and that this phenotype can be rescued by transgenic expression of the miRNA. Moreover, we demonstrate that *miR-989* is active in the somatic cells of the egg chamber and required in border cells for efficient migration. Predicted targets encompass most of the pathways known to be involved in regulation of border cell migration.

## Results and Discussion

Deep sequencing of an ovarian small RNA library identified *miR-989* as the most abundant miRNA species in the *Drosophila* ovary, constituting 15.9% of all annotated sequencing reads [18]. To test whether *miR-989* has an important function during oogenesis, we generated a deletion allele (designated *miR-989^KO^*) by ends-out homologous recombination [19], [20]. Deletion of the *miR-989* gene was confirmed by PCR on genomic DNA (not shown). Ovaries derived from young females bearing the *miR-989^KO^* allele *in trans* to a genomic deficiency (*Df(2R)Exel7130*) uncovering the *miR-989* locus proved to be morphologically normal (not shown).

### Delayed border cell migration

**Figure 2:**
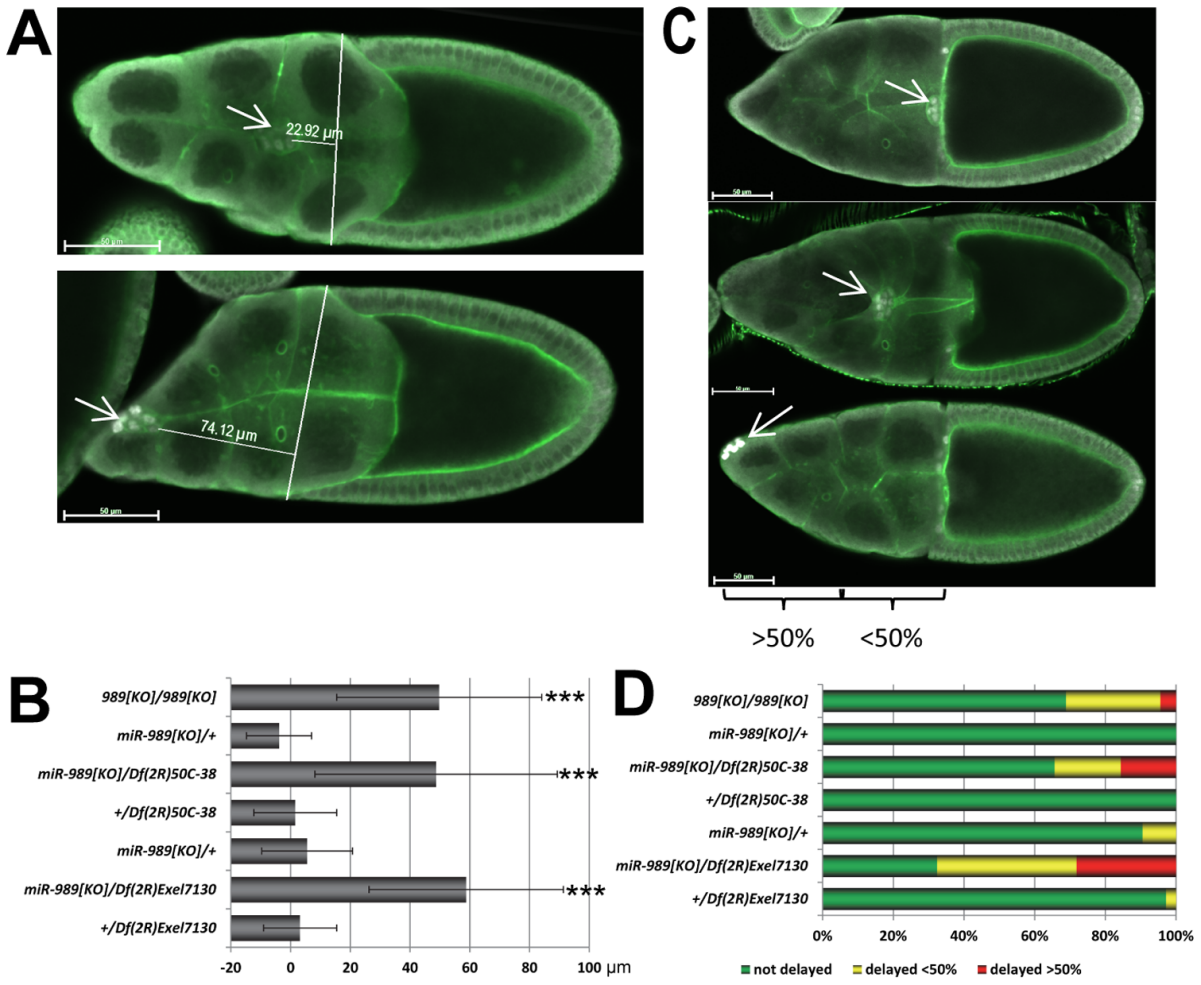
Border cell migration is delayed in *miR-989* mutant egg chambers. A Late stage 9 egg chambers, labelled with α-Slbo (white) and Phalloidin (green). Border cell clusters are highlighted by arrows. In *miR-989* mutant egg chambers, border cells were frequently delayed relative to the migrating main body follicular epithelium. B Quantification of the border cell migration phenotype in stage 9 and stage 10A egg chambers. Border cell migration is delayed in *miR-989* mutant egg chambers compared to heterozygous control egg chambers (*** p<0.001). X-Axis labels are in μm, error bars denote standard deviation. C Stage 10B egg chambers, labelled with α-Slbo (white) and Phalloidin (green). Border cell clusters are highlighted by arrows. Frequently, border cells had not reached the oocyte by this stage in *miR-989* mutant egg chambers. D Quantification of the border cell migration phenotype in stage 10B egg chambers.

We observed that border cell migration was frequently delayed in *miR-989^KO^* / *Df(2R)Exel7130* ovaries compared to controls and quantitated this phenotype during two stages of egg chamber development (Fig 2). During late stage S9 and S10A, we measured the distance between the leading border cell and the anterior-most cells in the sheet of follicle cells, as it migrates toward the oocyte (Fig. 2A). In all control genotypes, border cells kept pace with the advancing sheet of external follicle cells (Fig. 2B). In contrast, the border cell cluster lagged behind the follicular epithelium in homozygous *miR-989^KO^* egg chambers (p<0.001 in comparison to the heterozygous control). Similar results were obtained when the *miR-989^KO^* allele was placed in trans to two independent genomic deficiencies (*Df(2R)50C-38* and *Df(2R)Exel7130*) that uncover the *miR-989* locus (p<0.001 compared to all controls, Fig 2B). In wild type egg chambers, border cells typically have reached the oocyte by stage 10B and have begun to migrate toward their final anterodorsal position. In S10B egg chambers lacking *miR-989*, border cells were frequently found within the nurse cell compartment. We quantitated this phenotype by scoring whether the border cells had reached the oocyte (“not delayed”), or whether they were still found in the anterior half (“>50% delayed”) or posterior half (“<50% delayed”) of the nurse cell compartment (Fig. 2C). Using this scoring scheme, we found that most border cell clusters in the heterozygous control egg chambers had arrived at the oocyte at stage 10B. Over 1/3 of border cell clusters derived from *miR-989* mutant females were delayed. These observations suggested that *miR-989* is required for some aspect of border cell migration towards the oocyte.

### *miR-989* activity in somatic cells

**Figure 3:**
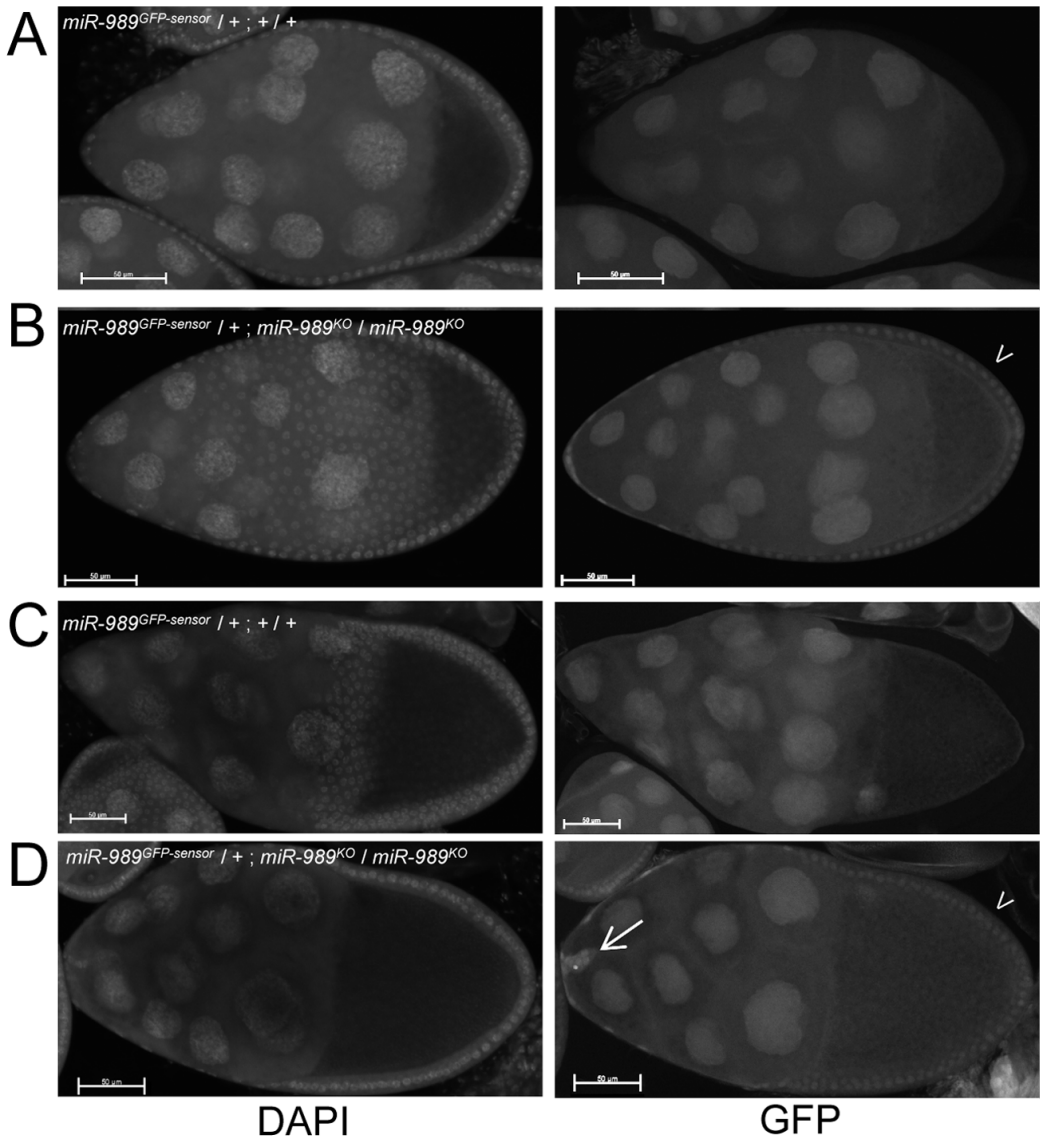
A miRNA sensor suggest somatic miR-*989* activity. Stage 8 (A, B) and Stage 10A (C, D) eggchambers expressing a *miR-989* GFP sensor in a wild type (A, C) or in a *miR-989* mutant background (B, D). While the GFP sensor was expressed at similar levels in the germ line cells in both genetic backgrounds, it was not detectable in the somatic follicle cells in the wild-type egg chambers. However, follicle cells were positively labelled by the sensor in the absence of *miR-989*. Note that the sensor also labels the border cells in (D) (arrow).

Border cell migration depends on guidance signals from germ-line cells, signal interpretation by the border cells to produce directed migration and interaction between the two cell types to allow movement of the border cell cluster on and between the nurse cells [5], [6], [7]. We therefore asked whether *miR-989* was acting in the somatic cells or germ line cells of the ovary. As a first step to address this question, we generated transgenic flies that express a miRNA sensor [21] for *miR-989*. This sensor encodes eGFP expressed ubiquitously under control of a tubulin promoter, followed by a 3' UTR containing two target sites for *miR-989*. In a wild type background, sensor-directed GFP was present in all the germ line cells, but GFP was not detected in the somatic follicle cells (Fig. 3 A, C). In the *miR-989* mutant background, a homogenous GFP signal was also observed in all somatic cells, including the border cell cluster while GFP expression in the germ line cells was unchanged (Fig. 3B, D, arrows). This suggests that *miR-989* is predominantly active in the somatic follicle cells.

### *miR-989* is required in somatic cells for efficient border cell migration

**Figure 4:**
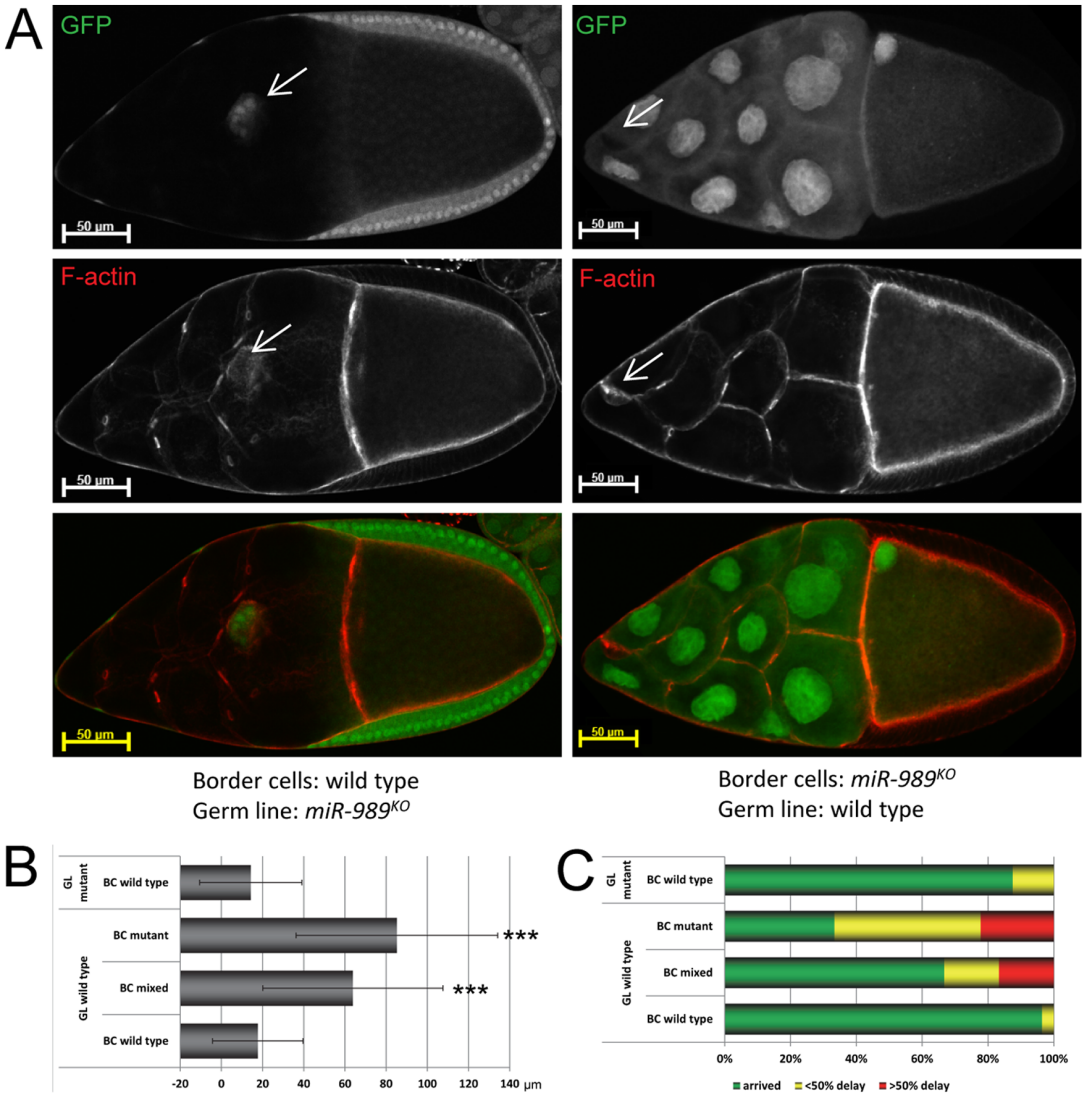
Clonal analysis demonstrates a somatic requirement for *miR-989* for normal border cell migration. A Genetic mosaics: wild type cells are labelled by nuclear GFP (upper panel) while *miR-989* mutant cells do not express GFP. The middle panels are labelled with phalloidin to highlight the F-actin cytoskeleton. The two egg chambers shown represent extreme cases of mosaicism. In the stage 10A egg chamber on the left all somatic cells are wild-type (GFP positive), while all germ line cells are mutant for *miR-989* (GFP negative). The border cells were not delayed in their migration. In the stage 10A egg chamber shown on the right all germ line cells are wild type (GFP positive) while all somatic cells are mutant for *miR-989* (GFP negative). Border cell migration was strongly delayed. B Quantification of the migration defects in migrating border cells in late stage 9 and stage 10A egg chambers. GL denotes germ line, and BC border cells. Migration was strongly delayed if the border cells were mutant, but not if the germ line cells lacked *miR-989*. We observed a population of border cell clusters that were partially wild-type and partially mutant for *miR-989*. In these cases, border cell migration was also delayed in comparison to controls. *** indicates p<0.001. C Quantification of the migration defects in stage 10B egg chambers. Wild type border cells migrated normally when the germ line was mutant for *miR-989.* Conversely, *miR-989* mutant border cells were delayed when the germ line was wild type.

The predominant pattern of follicle cell expression suggested that *miR-989* activity may be required in the follicle cells or the border cells to promote normal border cell migration. To test this idea, we generated mosaic egg chambers that were partially wild-type and partially mutant for *miR-989* and scored the migratory behaviour of the border cells. We found that wild-type border cells migrated normally when the germ line cells lacked *miR-989* (Fig. 4A, left panel). In contrast, migration was delayed when somatic cells including the border cell cluster were mutant for *miR-989* but the germ line was wild-type (Fig. 4B, right panel). The quantification of these observations is shown in Figure 4(B, C). Border cell migration was delayed in a statistically significant manner (p<0.001) when all cells in the border cell cluster were mutant for *miR-989*, but the germ cells were wild-type. We did not observe a statistically significant delay in egg chambers in which the border cells were wild-type but the germline was mutant for *miR-989*. These results provide evidence that *miR-989* is required in somatic cells for normal migration, but dispensable in the germ-line.

We also observed egg chambers in which the border cell cluster was partially wild type and partially mutant for *miR-989* while germ line was wild-type (Fig. 4B, C). Intriguingly, such mixed genotype border cell clusters were also delayed in their migration (p<0.001, compared to control egg chambers). The delay was similar in magnitude to that of completely mutant clusters (p>0.1 between completely and partially mutant border cell clusters). This suggests that lack of *miR-989* in just some border cells is sufficient to cause delays affecting the entire cluster. In other words, the presence of wild-type border cells cannot compensate for the lack of *miR-989* in some cells. However, we do not exclude the possibility that loss of *miR-989* from other somatic cells might contribute to the border cell migration phenotype.

### The border cell migration defect can be rescued by transgenic *miR-989* expression

**Figure 5:**
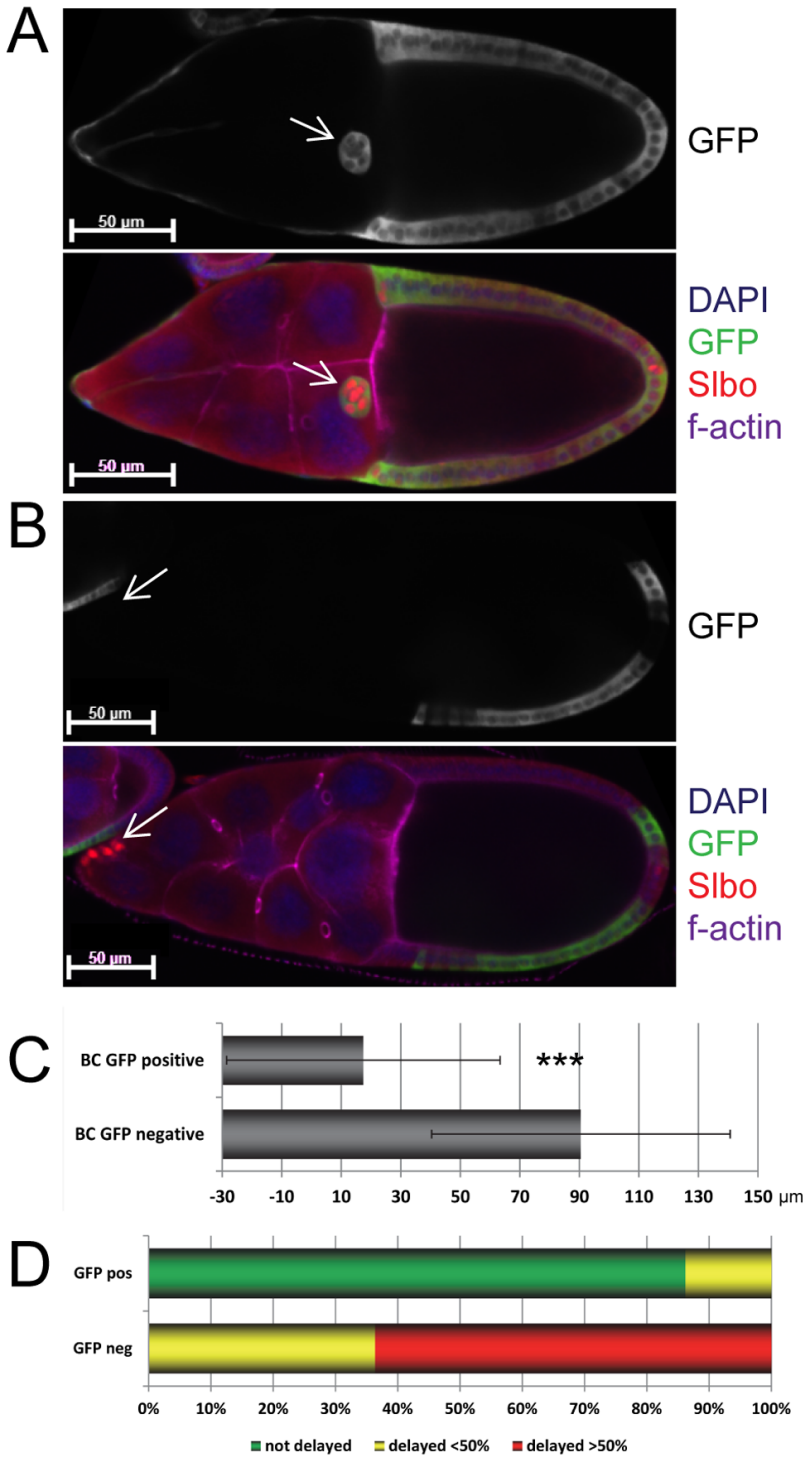
The border cell migration phenotype was rescued by transgenic expression of *miR-989.* A–D Expression of *miR-989* in border cells in a *miR-989* mutant background suppressed the delayed border cell migration phenotype. Presence of GFP indicates transgenic *miR-989* expression. A Example of a mosaic egg chamber in which *miR-989* and GFP were expressed in border cells and border cell migration was normal. B Example of a mosaic egg chamber in which *miR-989* and GFP were not expressed in border cells, and border cell migration was drastically delayed. C Quantification of border cell migration progress in mosaic stage 9 and stage 10A egg chambers. Mosaic egg chambers in which border cells were mutant for *miR-989* were strongly delayed in their migration. Transgenic expression of *miR-989* in border cells of sibling mosaics partially suppressed the delayed border cell phenotype. D Quantification of border cell migration progression in mosaic stage 10B egg chambers. Border cells transgenically expressing *miR-989* in a *miR-989* mutant background most often reached the oocyte by this stage. In contrast, *miR-989* and GFP negative border cells in sibling mosaics were strongly delayed.

To confirm that lack of *miR-989* was responsible for the border cell migration defects described above, we expressed *miR-989* from an UAS transgene. Expression with the *Slbo-Gal4* driver did not rescue border cell migration in a *miR-989^KO^* mutant background. In contrast, *miR-989* expression under the control of a heat-shock inducible *actin-flip-out-Gal4* cassette was able to rescue. This technique allowed us to restore miRNA expression in subsets of cells that were positively marked by presence of GFP. GFP-negative border cell clusters lacking *miR-989* were delayed in their migration (Fig. 5A, C, D), whereas GFP-positive border cell clusters with transgenic *miR-989* expression migrated almost normally (Fig. 5B, C, D; p<0.001). This demonstrates that loss of *miR-989* was responsible for the delayed border cell migration phenotype in the *miR-989* mutant egg chambers.

*miR-989* is expressed in the somatic cells throughout egg chamber development. In the flip-out clonal experiment, Gal4 expression was induced well before the onset of border cell migration. The *Slbo* gene turns on in stage 8, shortly before border cell migration begins. Expression of a miRNA under *Slbo-Gal4* control is expected to take some time to effectively repress its targets. Comparing the *Slbo-Gal4* and flip-out clonal rescue results suggests that *miR-989* may be required from the onset of border cell migration.

### predicted *miR-989* targets

*miR-989* target predictions were obtained from TargetScanFly [22], TargetScanFly ORF [3], MinoTar [3] and miRNA.org [23]. Together, these algorithms identify 724 non-redundant candidate target transcripts. We performed process GO term analysis using the GOrilla interface [24] to identify targets that might be involved in regulating border cell migration (Figure S1 and Table S1). This analysis revealed 163 enriched GO terms. Among these were potentially informative GO term nodes with lower GO term hierarchy level which were significantly enriched: ‘cell migration’ (enriched 2.8 fold, p = 1.6×10^−7^, 31 putative target genes), ‘cell adhesion’ (enriched 2.6 fold, p = 7.4×10^−7^, 25 putative targets), ‘cell projection morphogenesis’ (enriched 2.4 fold, p = 8.6×10–5, 23 putative targets), ‘transmembrane receptor protein tyrosine kinase signaling pathway’ (enriched 2.7 fold, p = 2.9×10^−4^, 16 putative targets) and ‘response to ecdysone’ (enriched 4.6 fold, p = 6.3×10^−4^, 7 putative targets). miRNAs typically downregulate their targets, in part through target transcript destabilization. It is therefore expected that target levels would increase in miRNA mutant cells. The ovary has two main cell types: somatic and germ line. Somatic cells, where *miR-989* is expressed, comprise a small fraction of the total tissue. Because the border cells are only a small fraction of the somatic cells, we have not attempted to validate upregulation of candidate targets in the *miR-989* mutant border cells by monitoring target RNA levels.

Predicted *miR-989* targets associated with the GO terms ‘cell migration’ and ‘regulation of cell projections’ are obvious candidates whose de-regulation may cause border cell migration phenotypes. The receptor tyrosine kinase receptors EGFR and Pvr are required in the border cells to receive guidance queues that direct cluster migration towards the oocyte [15], [16]. Upregulation of negative regulators of these signalling pathways could delay border cell migration. Ecdysone signaling play a crucial role in regulating the timing of border cell specification, and defects in border cell specification can delay migration [12], [13], [14].

Predicted *miR-989* targets associated with the GO term ‘cell adhesion’ may be particularly interesting. Border cells migrate collectively in a coordinated fashion, and migration events critically depend on remodelling events that change the adhesive properties of the migrating cells [5], [6], [7]. In the case of the border cell cluster, border cells have to give up their connection to neighbouring follicular epithelial cells when they initiate migration towards the oocyte. Likewise, while they do need to adhere to the nurse cell membranes, through homophilic interactions of DE-cadherin [17] they must not adhere too tightly in order to migrate efficiently. In this light, it is interesting to note that loss of *miR-989* from a subset of border cells is sufficient to cause border cell migration delays. Since border cells adhere tightly to each other [5], [6], [7], it is easily conceivable that few cells that adhere too tightly to the follicular epithelium or the migration substrate would impair migration of the entire cluster. This has been documented for clusters partially mutant for Slbo [25], which affects border cell specification and for clusters partially mutant for DE Cadherin [17]. *miR-989* could act on multiple targets including those affecting the processes discussed above to permit normal border cell migration.

## Materials and Methods

### Fly stocks

The *miR-989^KO^* allele was generated using pRMCE as described [19], [20]. The *UAS-miR-989* transgene was cloned into pUAST.attB-SLIC [26] and integrated into the landing site 86Fb to generate pT-989@Fb. To generate the *miR-989* sensor, oligos encoding two perfect *miR-989* binding sites were annealed and cloned into the tub>eGFP transgene [21]. The sensor was then transformed by P-element mediated germ line transformation and individual transgenic lines were established by standard procedures. Oligonucleotide sequences are available on request. The genomic deficiencies *Df(2R)Exel7130* and *Df(2R)50C-38* and the *hsFLP; FRT42A ubiGFPnls* strain were obtained from the Bloomington stock centre. *Actin-Flipout-Gal4 (AFG) UAS-10xGFP* was obtained from the Rørth lab [27]. For mosaic analysis (Fig. 4), males bearing a recombinant *FRT42A miR-989^KO^* chromosome were crossed to *hsFLP; FRT42A ubiGFPnls* virgins and offspring third instar larvae were heat-shocked for 1-2h at 37°C. For the rescue analysis, third instar larvae of the genotype *hsFLP/+; miR-989^KO^/miR-989^KO^; pT-989@Fb/AFG,10xGFP* were heat-shocked at 37°C for 1–2h and ovaries were then dissected from 2–3d old adult females for analysis.

### Immunostainings and Microscopy

1–3d old mated females were collected and placed on wet yeast for 12–24 h prior to dissection. Ovaries were dissected into Schneider's S2 cell medium supplemented with 50 μl/ml FCS and 1ul 10mg/ml Insulin (Sigma) per ml medium. Following dissection in 4% formaldehyde, samples were rinsed and washed in PBST (PBS +0.3% Triton-X), then blocked in PBSTB (PBST +3% BSA) and incubated overnight in primary antibody (rat anti slbo 1∶500) at 4°C. The ovaries were then extensively washed with PBSTB at room temperature and incubated overnight in secondary antibodies (AlexaFluor antibodies (Invitrogen), 1∶250 of the 1∶1 glycerol stocks). If applicable, Alexa633- or Alexa555-conjugated Phalloidin was added at 1∶150 (Invitrogen). Samples were then washed extensively and counterstained with DAPI. For sensor analysis, ovaries were fixed as above, extensively washed in PBST and counterstained with DAPI. Images were obtained either on a Zeiss Axio Imager 2.0 or a Zeiss LSM700 confocal microscope. Images were analysed with Zeiss Axiovision software. Graphs and statistical analysis were done with Microsoft Excel.

### GO term analysis of predicted *miR-989* targets

*miR-989* target lists were obtained from TargetScanFly [22], TargetScanFly ORF [3], MinoTar [3] and miRNA.org [23], standardized to CG gene name annotation using Flybase [28], pooled and then pruned of redundant entries using Excel. A genome-wide list of CG annotated genes in *Drosophila melanogaster* was obtained from Flybase as a reference pool. GO term analysis was performed using the GOrilla platform (http://cbl-gorilla.cs.technion.ac.il/), querying all *Drosophila* ontologies using the *miR-989* target predictions as target set and all *Drosophila* genes as a background set.

## Supporting Information

Figure S1 **GO term analysis of predicted** ***miR-989*** **targets.** Figure S1 shows a directed acyclic graph created by the GOrilla interface. It shows GO term enrichment of predicted *miR-989* targets (color coded).
(JPG)

Table S1 **GO terms enriched among the predicted** ***miR-989*** **targets.** Table S1 shows an annotated list of the GO terms that are enriched among predicted *miR-989* targets.
(XLSX)

## References

[1] Bartel DP (2009) MicroRNAs: target recognition and regulatory functions. Cell 136: 215–233. 19167326 10.1016/j.cell.2009.01.002 PMC3794896

[2] Bushati N, Cohen SM (2007) microRNA functions. Annu Rev Cell Dev Biol 23: 175–205. 17506695 10.1146/annurev.cellbio.23.090506.123406

[3] Schnall-Levin M, Zhao Y, Perrimon N, Berger B (2010) Conserved microRNA targeting in Drosophila is as widespread in coding regions as in 3'UTRs. Proc Natl Acad Sci U S A 107: 15751–15756. 20729470 10.1073/pnas.1006172107 PMC2936641

[4] Stark A, Brennecke J, Russell RB, Cohen SM (2003) Identification of Drosophila MicroRNA targets. PLoS Biol 1: E60. 14691535 10.1371/journal.pbio.0000060 PMC270017

[5] Montell DJ (2003) Border-cell migration: the race is on. Nat Rev Mol Cell Biol 4: 13–24. 12511865 10.1038/nrm1006

[6] Rorth P (2009) Collective cell migration. Annu Rev Cell Dev Biol 25: 407–429. 19575657 10.1146/annurev.cellbio.042308.113231

[7] Wu X, Tanwar PS, Raftery LA (2008) Drosophila follicle cells: morphogenesis in an eggshell. Semin Cell Dev Biol 19: 271–282. 18304845 10.1016/j.semcdb.2008.01.004 PMC2430523

[8] Bastock R, St Johnston D (2008) Drosophila oogenesis. Curr Biol 18: R1082–1087. 19081037 10.1016/j.cub.2008.09.011

[9] Beccari S, Teixeira L, Rorth P (2002) The JAK/STAT pathway is required for border cell migration during Drosophila oogenesis. Mech Dev 111: 115–123. 11804783 10.1016/s0925-4773(01)00615-3

[10] Silver DL, Geisbrecht ER, Montell DJ (2005) Requirement for JAK/STAT signaling throughout border cell migration in Drosophila. Development 132: 3483–3492. 16000386 10.1242/dev.01910

[11] Silver DL, Montell DJ (2001) Paracrine signaling through the JAK/STAT pathway activates invasive behavior of ovarian epithelial cells in Drosophila. Cell 107: 831–841. 11779460 10.1016/s0092-8674(01)00607-9

[12] Bai J, Uehara Y, Montell DJ (2000) Regulation of invasive cell behavior by taiman, a Drosophila protein related to AIB1, a steroid receptor coactivator amplified in breast cancer. Cell 103: 1047–1058. 11163181 10.1016/s0092-8674(00)00208-7

[13] Godt D, Tepass U (2009) Breaking a temporal barrier: signalling crosstalk regulates the initiation of border cell migration. Nat Cell Biol 11: 536–538. 19404334 10.1038/ncb0509-536

[14] Jang AC, Chang YC, Bai J, Montell D (2009) Border-cell migration requires integration of spatial and temporal signals by the BTB protein Abrupt. Nat Cell Biol 11: 569–579. 19350016 10.1038/ncb1863 PMC2675665

[15] Duchek P, Rorth P (2001) Guidance of cell migration by EGF receptor signaling during Drosophila oogenesis. Science 291: 131–133. 11141565 10.1126/science.291.5501.131

[16] Duchek P, Somogyi K, Jekely G, Beccari S, Rorth P (2001) Guidance of cell migration by the Drosophila PDGF/VEGF receptor. Cell 107: 17–26. 11595182 10.1016/s0092-8674(01)00502-5

[17] Niewiadomska P, Godt D, Tepass U (1999) DE-Cadherin is required for intercellular motility during Drosophila oogenesis. J Cell Biol 144: 533–547. 9971747 10.1083/jcb.144.3.533 PMC2132905

[18] Czech B, Malone CD, Zhou R, Stark A, Schlingeheyde C, et al (2008) An endogenous small interfering RNA pathway in Drosophila. Nature 453: 798–802. 18463631 10.1038/nature07007 PMC2895258

[19] Chen YW, Weng R, Cohen SM (2011) Protocols for use of homologous recombination gene targeting to produce microRNA mutants in Drosophila. Methods Mol Biol 732: 99–120. 21431708 10.1007/978-1-61779-083-6_8

[20] Weng R, Chen YW, Bushati N, Cliffe A, Cohen SM (2009) Recombinase-mediated cassette exchange provides a versatile platform for gene targeting: knockout of miR-31b. Genetics 183: 399–402. 19564483 10.1534/genetics.109.105213 PMC2746163

[21] Brennecke J, Hipfner DR, Stark A, Russell RB, Cohen SM (2003) bantam encodes a developmentally regulated microRNA that controls cell proliferation and regulates the proapoptotic gene hid in Drosophila. Cell 113: 25–36. 12679032 10.1016/s0092-8674(03)00231-9

[22] Ruby JG, Stark A, Johnston WK, Kellis M, Bartel DP, et al (2007) Evolution, biogenesis, expression, and target predictions of a substantially expanded set of Drosophila microRNAs. Genome Res 17: 1850–1864. 17989254 10.1101/gr.6597907 PMC2099593

[23] Betel D, Wilson M, Gabow A, Marks DS, Sander C (2008) The microRNA.org resource: targets and expression. Nucleic Acids Res 36: D149–153. 18158296 10.1093/nar/gkm995 PMC2238905

[24] Eden E, Navon R, Steinfeld I, Lipson D, Yakhini Z (2009) GOrilla: a tool for discovery and visualization of enriched GO terms in ranked gene lists. BMC Bioinformatics 10: 48. 19192299 10.1186/1471-2105-10-48 PMC2644678

[25] Rorth P, Szabo K, Texido G (2000) The level of C/EBP protein is critical for cell migration during Drosophila oogenesis and is tightly controlled by regulated degradation. Mol Cell 6: 23–30. 10949024 10.1016/s1097-2765(05)00008-0

[26] Szuplewski S, Kugler JM, Lim SF, Verma P, Chen YW, et al (2012) MicroRNA transgene overexpression complements deficiency-based modifier screens in Drosophila. Genetics 190: 617–626. 22095085 10.1534/genetics.111.136689 PMC3276628

[27] Poukkula M, Cliffe A, Changede R, Rorth P (2011) Cell behaviors regulated by guidance cues in collective migration of border cells. J Cell Biol 192: 513–524. 21300853 10.1083/jcb.201010003 PMC3101089

[28] McQuilton P, St Pierre SE, Thurmond J (2012) FlyBase 101–the basics of navigating FlyBase. Nucleic Acids Res 40: D706–714. 22127867 10.1093/nar/gkr1030 PMC3245098

