# Correction: *miR-989* Is Required for Border Cell Migration in the *Drosophila* Ovary

**DOI:** 10.1371/annotation/709181e7-dfc9-40e9-bd0b-bd1c5541c2b1

**Published:** 2013-07-10

**Authors:** Jan-Michael Kugler, Ya-Wen Chen, Ruifen Weng, Stephen M. Cohen

Pushpa Verma's name was inadvertently omitted in the list of author list for the article. The authors would like to revise the author list to read as below:

Jan-Michael Kugler, Pushpa Verma, Ya-Wen Chen, Ruifen Weng, Stephen M.Cohen

Pushpa Verma 's affiliation is Institute of Molecular and Cell Biology, Singapore. 

