# Supplementary figures and images for: miR-989 Is Required for Border Cell Migration in the Drosophila Ovary
Source: PLoS One. 2013 Jul 3;8(7):e67075. doi: 10.1371/journal.pone.0067075 (PMC3700948; doi:10.1371/journal.pone.0067075)

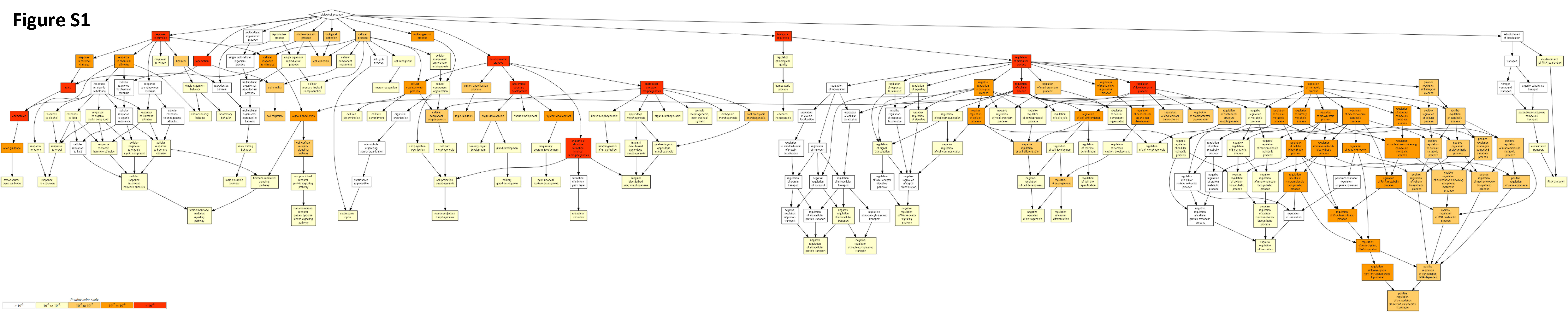

Supplement: Figure S1 — GO term analysis of predicted miR-989 targets. Figure S1 shows a directed acyclic graph created by the GOrilla interface. It shows GO term enrichment of predicted miR-989 targets (color coded). (JPG) [file pone.0067075.s001.jpg]
